# A population-based study of Kaposi Sarcoma-associated herpesvirus seropositivity in Uganda using principal components analysis

**DOI:** 10.1186/1750-9378-8-3

**Published:** 2013-01-16

**Authors:** Joanne T Chang, Fatma M Shebl, Ruth M Pfeiffer, Benon Biryahwaho, Barry I Graubard, Sam M Mbulaiteye

**Affiliations:** 1 Division of Cancer Epidemiology and Genetics, National Cancer Institute, National Institutes of Health, Department of Health and Human Services, Bethesda, MD, USA; 2 Uganda Virus Research Institute, Entebbe, Uganda; 3 University of Michigan, School of Public Health, Ann Arbor, MI, USA; 4 Currently at Yale School of Public Health, New Haven, CT, USA

**Keywords:** Kaposi sarcoma-associated herpesvirus, Uganda, Kaposi sarcoma, Socioeconomic, Principal Components Analysis, Human herpesvirus 8

## Abstract

**Background:**

Kaposi sarcoma-associated herpesvirus (KSHV) seropositivity is associated with sexual, environmental, and socioeconomic exposures. Whether these characteristics are independent risk factors is uncertain because of reliance on selected high-risk or hospital-based populations and incomplete adjustment for confounding. Therefore, we evaluated risk factors for KSHV seropositivity in a population-based study in Uganda using principal components analysis (PCA).

**Methods:**

The study population comprised 2,681 individuals randomly selected from a nationally-representative population-based HIV/AIDS sero-behavioral survey conducted in 2004/05. Questionnaire and laboratory data (97 variables) were transformed into a smaller set of uncorrelated variables using PCA. Multivariable logistic regression models were fitted to estimate odds ratios and 95% confidence intervals for the association between components and KSHV seropositivity.

**Results:**

Data were reduced to three principal components (PCs) labeled as Sexual behavioral, Socioeconomic, and Knowledge PCs. In crude analysis, KSHV seropositivity was associated with the Knowledge (*p*_*trend*_ = 0.012) and Socioeconomic components (*p*_*trend*_ = 0.0001), but not with the Sexual-behavioral component (*p*_*trend*_ = 0.066). KSHV seropositivity was associated with the Socioeconomic PC (*p*_*trend*_ = 0.037), but not with the Sexual-behavioral and Knowledge PCs, in the models including PCs, age, gender and geographic region.

**Conclusions:**

Our results fit with the view that in Uganda socioeconomic characteristic may influence KSHV seropositivity. Conversely, the results fit with the interpretation that in Uganda sexual-behavioral characteristics, if relevant, contribute minimally.

## Background

Kaposi sarcoma-associated herpesvirus (KSHV) also called Human herpesvirus 8 (HHV-8), discovered in 1994 in Kaposi sarcoma (KS) tissue from an AIDS patient [1], is the infectious cause of KS [2]. In addition to KS, KSHV also is linked to primary effusion lymphoma (PEL), a rare aggressive serous cavity lymphoma [3], and to Castleman’s disease, a benign lymphoproliferative disorder [4]. In 2010, KSHV was declared a Group 1 carcinogenic agent, highlighting its public health significance in countries where it is endemic [5].

KSHV seropositivity is highest (>50-80%) in equatorial Africa [6,7], including in Uganda, where 1 in every 2 persons is KSHV seropositive by adult age [8,9]. KSHV seropositivity is intermediate (10%) in Mediterranean Europe [10] and rare (<3%) in the North America and Northern Europe [11,12], except among homosexual men in whom up to 1 in every 3 men may be KSHV seropositive, most likely due to transmission via sexual contact [13]. In KSHV endemic regions, KSHV transmission is associated with casual contact between an infected mother, sibling, or other family member [14-16], particularly in children, and with poor socioeconomic status [14]. Transmission may occur via contact with KSHV in saliva in crowded poor households [15]. Interestingly, KSHV seropositivity increases with age in endemic regions, indicating that sexual transmission may occur, although this might also be due to virus reactivation later in life increasing detection of antibodies [17]. KSHV seropositivity has been positively associated with some sexual risk factors such as number of marital unions and number of children born in some studies [8,18], but not with other sexual risk factors such as ever had other STIs in other studies [19,20]. The validity and/or independence of associations of KSHV seropositivity with sexual, socioeconomic, and family characteristics is uncertain because those findings were based on studies conducted in hospital-based populations [21], individuals with sexually transmitted diseases or commercial sex workers [18,20,22] or selected occupational groups [23], whose results may not be generalized.

We previously showed statistically significant positive association between KSHV seropositivity and male gender, increasing age, low or no formal education, and residence in a KS endemic geographical area in Uganda [8], using data from a nationally representative population-based sample of adults in Uganda [8,9]. In a follow-up analysis of the same subjects, KSHV seropositivity was positively associated with number of marital unions, number of children born, and inversely associated with having ever used a condom [9], possibly implicating sexual transmission. However, KSHV seropositivity was not associated with human herpes simplex 2 (HSV2), human immunodeficiency virus (HIV) infections and other sexual variables, casting doubt on the significance of heterosexual sexual KSHV transmission in this population. Nonetheless, as this study evaluated only 18 of 97 variables for association with KSHV seropositivity, confounding or non-specificity of associations could not be excluded [8,23].

In this paper we conducted a principal components analysis (PCA), an agnostic data reduction method that allows us to construct underlying exposure components on the cohort using data from 97 variables collected in the original Uganda HIV/AIDS sero-behavioral survey (UHSBS) [24] in 2004/05 study to evaluate associations with KSHV seropositivity in Uganda.

## Results

We identified three independent principal components (PCs), which were labeled descriptively as Sexual behavioral, Socioeconomic, and Knowledge PCs (Table 1). Variables contributing to the “Sexual behavioral” PC included ever had received blood transfusion, tested for HIV positive, tested for HSV positive, number of children, being currently married, and not using a condom during the last sex intercourse. Variables contributing to the “Socioeconomic” PC included wealth index, non-mud based flooring material, higher education status, owning a radio, telephone, bicycle, or car (ownership of communication device or transportation method), having electricity access, and ownership of durable goods. Variables contributing to the “Knowledge” PC included can people avoid or reduce the chance of getting AIDS by avoid having sex with homosexual, getting mosquito bites, kissing and getting protection from traditional healer.

**Table 1:** **Factor loadings from principal components analysis of UHSBS data years in 2004-2005**^**a**^

	**Principal components**^**b**^		
**Variables**	**Sexual behavioral**	**Socioeconomic**	**Knowledge**
Have you ever had a blood transfusion?	**0.785**	0.013	−0.007
Number of children	**0.783**	−0.104	0.024
Are you HSV positive?	**0.777**	−0.031	0.013
Are you HIV positive?	**0.775**	0.029	0.013
Did you use condom during the last sex intercourse?	**0.731**	−0.066	0.050
Are you currently married?	**0.675**	−0.120	−0.003
Number of lifetime sex partners	**0.496**	0.038	0.001
Occupation	**0.404**	0.187	0.025
Wealth Index	−0.049	**0.748**	0.043
Use mud as floor material?	−0.126	**0.701**	0.002
Education level	−0.176	**0.594**	−0.009
Ownership of communication device	−0.027	**0.522**	0.068
Ownership of durable good?	−0.029	**0.505**	0.043
Electricity access?	−0.127	**0.487**	0.017
What type of toilet does your household have?	−0.090	**0.457**	0.011
Ethnicity-Baganda	0.031	**0.435**	−0.062
Use of bednet?	−0.047	**0.402**	0.014
Would you buy vegetables from a vendor had AIDS?	0.053	**0.401**	−0.058
Have you heard of any drugs that can PROLONG THE LIFE of a person who has the virus?	0.165	**0.383**	−0.068
Are there any special drugs that a doctor or nurse can give to pregnant women infected with AIDS to reduce the risk of transmission?	0.099	**0.370**	−0.051
Should a HIV-infected female teacher be allowed to teach in school?	0.128	**0.362**	−0.057
Source of water?	−0.015	**0.352**	−0.008
^c^By avoiding sex with homosexual?	0.022	−0.140	**0.909**
^c^By avoiding mosquito bites?	−0.016	0.120	**−0.798**
^c^By avoiding kissing?	−0.031	0.098	**−0.845**
^c^By protection from traditional healer	−0.030	0.149	**−0.898**
By avoiding partners who have many partners?	−0.020	−0.075	**0.687**
By avoiding sex prostitute?	0.035	−0.068	**0.682**
By avoiding blood transfusions?	−0.047	0.055	**0.587**
By asking partner to get tested?	−0.010	0.011	**0.495**
By avoiding injections?	−0.054	0.021	**0.446**
By limit number of sex partners?	0.085	0.007	**0.418**
By avoid sharing razor blades with AIDS patients?	−0.151	0.085	**0.311**
Are you a Christian?	−0.020	−0.255	0.051
Are you a Muslim?	0.001	0.265	−0.060
Are you currently divorced?	0.048	−0.031	0.001

^a ^Only showing 35 out of 74 variables; ^b ^These components were labeled descriptively to reflect variables with high component loadings; ^c^ Can people avoid or reduce the chance of getting AIDS by these routes.

In unadjusted analyses (Table 2), KHSV seropositivity was significantly associated with Socioeconomic (*p*_*trend*_ = 0.0001) and Knowledge (*p*_*trend*_ = 0.012) PCs, but it was not associated with the Sexual behavioral PC (*p*_*trend*_ = 0.07). Higher scores on the Socioeconomic and Knowledge PCs were associated with lower KSHV seropositivity. The association of KSHV seropositivity with the Socioeconomic (*p*_*trend*_ = 0.002) and Knowledge (*p*_*trend*_ = 0.018) PCs remained significant when we adjusted for age, sex and geographical area of residence. The category-specific ORs between KSHV seropositivity and the Knowledge and Sexual behavioral, but not Socioeconomic, PCs were substantially attenuated or the effect was reversed in some cases when we adjusted associations for the PCs, suggesting that the crude associations with these PCs were confounded. KSHV seropositivity was significantly associated with the Socioeconomic PC, but not with Knowledge PC when the PCs were entered into a multivariable model that also included age, sex, and geographical region (*p*_*trend*_ =0.038). When we tested for goodness-of-fit, a multivariable including the Socioeconomic PC fit the data better than models including the Knowledge and Sexual behavioral PCs (log-likelihood p < 0.0001).

**Table 2:** Seroprevalence and association between KSHV seropositivity and Principal components (PCs) by quartile

**Principal components**	**KSHV seropositivity**		**Univariate**		**Multivariable**^**a**^		**Full multivariable model**^**b**^	
	**%**	**95% CI**	**OR**	**95% CI**	**OR**	**95% CI**	**OR**	**95% CI**
**Sexual behavioral**								
1^st^ quartile	49.2	50.0, 54.4	Ref	Ref	Ref	Ref	Ref	Ref
2^nd^ quartile	56.6	51.5, 61.6	1.35	1.01, 1.80	1.11	0.82, 1.51	1.08	0.79, 1.46
3^rd^ quartile	56.7	51.9, 61.7	1.36	1.02, 1.80	1.06	0.77, 1.45	1.02	0.73, 1.42
4^th^ quartile	56.2	51.5, 60.9	1.33	1.01, 1.73	0.95	0.69, 1.32	0.96	0.68, 1.35
*P value* for heterogeneity	*0.106*		*0.10*		*0.74*		*0.89*	
*P value* for trend			*0.066*		*0.61*		*0.58*	
**Socioeconomic**								
1^st^ quartile	61.6	57.3, 65.9	Ref	Ref	Ref	Ref	Ref	Ref
2^nd^ quartile	56.8	52.2, 61.4	0.82	0.65, 1.05	0.85	0.67, 1.08	0.87	0.67, 1.14
3^rd^ quartile	48.8	43.7, 53.9	0.60	0.46, 0.78	0.61	0.47, 0.81	0.68	0.49, 0.92
4^th^ quartile	50.6	45.4, 55.8	0.64	0.49, 0.84	0.67	0.50, 0.91	0.78	0.54, 1.13
*P value* for heterogeneity	0.0003*		0.0004*		*0.005*		*0.095*	
*P value* for trend			0.0001*		0.002*		0.037*	
**Knowledge**								
1^st^ quartile	59.9	55.5, 64.4	Ref	Ref	Ref	Ref	Ref	Ref
2^nd^ quartile	55.5	50.0, 60.9	0.83	0.63, 1.11	0.85	0.64, 1.14	1.00	0.74, 1.36
3^rd^ quartile	47.7	42.8, 52.5	0.61	0.47, 0.79	0.63	0.48, 0.82	0.76	0.57, 1.02
4^th^ quartile	54.4	49.8, 58.9	0.80	0.62, 1.02	0.80	0.63, 1.03	0.96	0.74, 1.25
*P value* for heterogeneity	0.004*		0.003*		0.006*		*0.18*	
*P value* for trend			0.012*		0.018*		*0.49*	

^a. ^Adjusted for age, sex, geographical region; ^b. ^Adjusted for age, sex, and geographical region and other PCs; * *p-value* < 0.0.

## Discussion

We found that in Uganda, where KSHV is highly endemic, KSHV seropositivity was inversely associated with high factor scores on the Socioeconomic and Knowledge PCs, but not with Sexual behavioral PC. The association remained significant when we adjusted for sex, age, and geographical regions, which were considered confounders of KSHV seropositivity in this population [12], suggesting that the findings may be valid.

Our study results are generally consistent with previous studies, which have suggested role of socioeconomic status in KSHV seropositivity in Uganda, based on a study which also used a factor analysis approach [14]. In that study, KSHV seropositivity was inversely associated with low factor scores of maternal and environmental factors, which are surrogates for socioeconomic status. However, that study was based on hospital-based population and relatively limited number of covariates that was available for adjustment. Our results, from a much larger population-based sample that is geographically representative of the Ugandan population, confirm the role of socioeconomic status in KSHV seropositivity in Uganda. The null association with the Sexual behavioral PC does not support an important role of sexual behavior on KSHV seropostivity in the general population in Uganda. This conclusion is different from the one reached by Shebl et al. [9] Although KSHV seropositivity was not associated with HIV or HSV2 infections, with lifetime number of sexual partners, having at least one sexually transmitted disease were unrelated to KSHV seropositivity, it was inversely associated with ever use of condom use and positively associated with being married and with each additional child born, suggested a possible, albeit, small role of sexual transmission. Our study found a marginal association between the Sexual behavior PC and KSHV seropositivity in crude analyses, but the association disappeared after adjusting for the other PCs, suggesting that the association detected with sexual variables may be due to confounding by other poorly understood socioeconomic factors.

While our results should not be interpreted as proving the lack of KSHV transmission via sexual contact, they support conclusions reached in other studies that that sexual transmission of KSHV is not a major source of infection in the general population [20,25,26]. KSHV seropositivity varies substantially by geography at a global as well local level. For example, de Sanjose and colleagues [26] observed significant variation in KSHV seroprevalence from 3.8% in Spain to 46% in Nigeria among women participating in a large international study conducted by the International Agency for Research on Cancer. KSHV seropositivity is lower in wealthier countries in Africa, such as South Africa, and higher in poorer countries, such as Uganda [25-27]. Within countries, KSHV seropositivity is higher in rural areas but lower in urban areas [28,29]. The biological basis of this variation is presently unclear to us. Our results suggest that variation in socioeconomic status may play a role in the geographical variation of KSHV seropositivity [14,26,28]. We previously hypothesized that infection with stool parasites, which is highly prevalent in poor countries, may play a role in KSHV transmission [30,31]. Findings by Lin et al. that KS patients attending the Uganda Cancer Institute were more likely to have a higher carriage of *Strongyloides* parasites than patients with other cancers treated at the same hospital [30] provide some support for the hypothesis. The hypothesis was also supported by a study of mother-infant pairs in Uganda [31], which reported that detection of malaria parasitaemia, hookworm and *Mansonella perstans* in stool was associated KSHV seropositivity. However, KSHV was not associated with some other parasites evaluated in the same study, including *Schistosoma mansoni, Strongyloides stercoralis, Trichuris trichiura, Ascaris lumbricoides and Trichostrongylus* species, suggesting that the role of parasites in the geographical variation of KSHV seropositivity requires further evaluation.

### Strengths and limitations

The strengths of our study include having detailed data on a nationally representative sample, which enabled us to use PCA methods to evaluate associations with KSHV seropositivity. Exclusion of variables where prior studies indicated significant associations enabled us to confirm those previous associations after taking into account all available data. PCA has the ability to identify independent factors that explain the maximum amount of mutual correlation [32]. However, our findings should be interpreted with caution. First, while the PCA methods offer a parsimonious way to transform many correlated variables into a few uncorrelated variables, it may mask relevant associations. For instance, some sexual behavioral variables such as ever used condom from the last sex intercourse or number of children born were found to be associated with KSHV seropositivity [9], but PCA does not reveal this association. Moreover, since PCA is summing up across errors in data collection, data entry and laboratory testing, some contradictory patterns can be found compared to previous findings. Second, our data are cross-sectional; therefore, it is not possible to determine the direction of causality. Third, KSHV serology is imperfect [33], thus, misclassification of results, although it would be random, is possible.

## Conclusions

To summarize, we report a significant association between low socioeconomic status and KSHV seropositivity. Sexual behavioral and Knowledge PCs were unrelated to KSHV seropositivity. Future work is needed to better understand how socioeconomic exposures influence KSHV transmission in countries where KSHV and KS are endemic.

## Materials and methods

### Ethical approval

The Uganda Virus Research Institute (UVRI) Science Ethics Committee and the National Council of Science and Technology in Uganda and from the Centers for Disease Control and Prevention (CDC, Atlanta, Georgia, USA) gave ethical approval to conduct the study.

### Study population

Details of this study have been previously reported [8,9,34,35]. Briefly, the study was based on a subset of data from the original 19,656 Ugandans aged 15–59 years enrolled the UHSBS in 2004–2005 to investigate national and subnational patterns of HIV/AIDS in Uganda [34,35]. The UHSBS used a two-stage cluster probability sample design to obtain a nationally representative sample of adults. In the first stage, 417 clusters were selected from census enumeration areas of the 2002 Uganda National Census [36]. In the second stage, 25 households were randomly selected per cluster. All adult residents in the household were invited to participate, for a total sample size of 10,437 individuals.

The KSHV studies are based on 2,681 individuals randomly selected from the original UHSBS sample and tested for KHSV serologically [8,9]. Detailed descriptions of the Uganda cohort and blood sampling technique have been described in previous studies [8,9].

### KSHV serology and testing

KSHV serology was performed at UVRI in Uganda using synthetic peptides encoded by K8.1 and orf65 viral genes manufactured at the CDC Herpes Virology Laboratory in Atlanta [37,38]. The qualities of KSHV serology at UVRI and the CDC were comparable, based on parallel testing of a panel of well characterized samples subjects with KS, asymptomatic Ugandans, and HIV positive and negative Caucasians provided by the National Cancer Institute (Spearman’s rank correlation coefficients for optical density (OD) values = 0.86 for K8.1 and OD = 0.78 for orf65, both *p* < 0.001). Although KSHV serology remains imperfect, the combined assays have a sensitivity of about 93% [38] and a specificity of about 98% [39] in African samples. KSHV results were categorized as positive when the adjusted K8.1 OD was >0.7 or 0.5-0.7 and the adjusted orf65 OD was >2.5, negative when the adjusted K8.1 OD was 0.5-0.7 and the adjusted orf65 OD was ≤2.5, and indeterminate when the adjusted K8.1 OD was 0.5-0.7 and the orf65 OD was <2.5.

### Data and variables

Detailed analyses of KSHV seropositivity patterns have been assessed and described in previous studies [8,9]. To observe the complete pattern of KSHV seropositivity in Uganda, this study included all questions were asked in the UHSBS including questions with knowledge and attitudes on HIV/AIDS and additional household questions, which was not elicited described in previous studies [8,9,34,35].

The data included information from household (18 variables) and individual (76 variables) questionnaires and from results of laboratory tests (3 variables: HIV, HSV2 (all subjects) and hepatitis B virus (HBV) (available on the same random1/3 subsample of subjects)). The household questionnaire elicited information on ownership of durable goods, car, motorcycle or bicycle, television, mobile phone and landline phone, mosquito bed net, source of drinking water (surface, well/public, well/private, piped/private), type of material used to build the floor, source of electricity, and type of toilet used (none, uncovered pit, covered pit, or flush toilet). The individual questionnaire elicited information on demographic information, knowledge and attitudes about how HIV may be transmitted or not and their knowledge about correct and incorrect routes of HIV/AIDS, their attitudes toward people who may have HIV/AIDS. For example, demographic information includes level of education attained, current occupation, ethnicity, religion, number of children born, and current marital status. Questions about knowledge and attitudes about HIV transmission include “can people avoid or reduce the chances of getting AIDS or the virus that causes AIDS by avoiding mosquito bites?”, or “can people avoid or reduce the chances of getting AIDS or the virus that causes AIDS by using condom during sex?”; questions about their attitudes toward people who may have HIV/AIDS include “would you take care of a relative who is living with AIDS?”, or “should a HIV-infected female teacher be allowed to teach in school?”.

### Statistical methods

Principal Components are linear combinations of the input variables and explain as much of the variation in the data as possible [32]. Large positive or negative factor loadings indicate the variable that is more representative in that component. Factor loadings with a magnitude at least 0.3 were used to describe the component.

**Figure 1:**
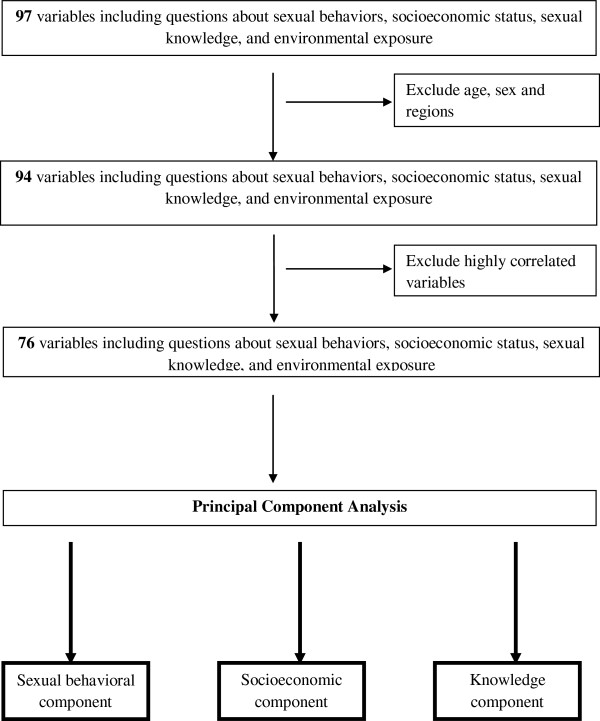
Flow chart showing data reduction approach applied to household- and individual-questionnaire variables and laboratory results during principal components analysis.

Statistical analyses were performed in SAS version 9.2 using PROC FACTOR procedure (SAS Institute, Cary, NC, USA). Analysis utilized available data from 97 variables collected during the UHSBS. PCA was utilized to transform the 97 correlated variables in the dataset into a smaller set of uncorrelated variables (Figure 1) [32]. Because age, gender, and geographic region were previously shown to be significantly associated with KSHV seropositivity [22], they were excluded from the PCA. To avoid redundancy, *Pearson* correlations of all pairs of 94 variables were computed, and when the correlation coefficients were greater than 0.90, only one of the two variables was chosen to be included in the PCA. The chosen variable was the one with less missing values. In addition, if the sample size were similar, variable with less correlation was selected. This resulted in exclusion of 21 variables. Thus, 76 representative variables were retained for PCA. The PCs were identified, based on the patterns of the Scree plots. Three PCs were identified from and the resulting PCs were transformed into uncorrelated through orthogonal rotation (varimax). The PCs were labeled descriptively based on the variables with high factor loadings (>0.3).

The association between PCs and KSHV seropositivity was evaluated by entering PCs, PC scores were computed for each individual and were divided into quartiles to create categorical variables. The odds ratios (ORs) and 95% confidence intervals (CIs) were used to estimate the association between KSHV seropositivity and the PCs. The logistic regression analyses were weighted for the sample and accounted for the cluster sampling of the UHSBS using the SAS survey procedure PROC SURVEYLOGISTIC. Age, gender, and geographic location, which were not included in the derivation of PCs but were considered *a priori* established risk factors for KSHV seropositivity [7], were entered as covariates in logistic regression to adjust for potential confounding of the association between PCs and KSHV seropositivity. 2-sided *p-*values were obtained using the *Rao-Scott* tests [40] were considered statistically significant if less than 0.05.

### Consent

Written informed consent was obtained for the patient for publication of this report.

## Abbreviations

KSHV: Kaposi sarcoma-associated herpesvirus; HHV8: Human herpesvirus 8; PCA: Principal components analysis; PC: Principal component; KS: Kaposi sarcoma; PEL: Primary effusion lymphoma; HSV2: Herpes simplex 2; HIV: Human immunodeficiency virus; UHSBS: Uganda HIV/AIDS sero-behavioral survey; UVRI: Uganda virus research institute; CDC: Centers for disease control and prevention; OD: Optical density OD; HBV: Hepatitis B virus; OR: Odds ratios; CI: Confidence interval.

## Competing interests

The authors declare that they have no competing interests.

## Authors’ contributions

All authors listed have contributed to the project. JC conducted the data analysis, interpreted data, and drafted the manuscript. FS helped design the study, supervised data analysis, and interpreted data. RP and BG supervised statistical analyses and interpreted data. BB performed KSHV serology and interpreted data. SM conceived the study, directed its implementation, interpreted data, and edited the manuscript. All authors reviewed, revised, and approved the manuscript.

## References

[B1] Chang Y Cesarman E Pessin MS Lee F Culpepper J Knowles DM Moore PS Identification of herpesvirus-like DNA sequences in AIDS-associated Kaposi’s sarcoma Science 1994 266 5192 1865 1869 10.1126/science.7997879 7997879

[B2] Martin JN Kaposi sarcoma-associated herpesvirus/human herpesvirus 8 and Kaposi sarcoma Adv Dent Res 2011 23 1 76 78 10.1177/0022034511399913 21441485

[B3] Cesarman E Chang Y Moore PS Said JW Knowles DM Kaposi’s sarcoma-associated herpesvirus-like DNA sequences in AIDS-related body-cavity-based lymphomas N Engl J Med 1995 332 18 1186 1191 10.1056/NEJM199505043321802 7700311

[B4] Soulier J Grollet L Oksenhendler E Cacoub P Cazals-Hatem D Babinet P D’Agay MF Clauvel JP Raphael M Degos L Kaposi’s sarcoma-associated herpesvirus-like DNA sequences in multicentric Castleman’s disease Blood 1995 86 4 1276 1280 7632932

[B5] IARC Monographs on the Evaluation of Carcinogenic Risks to Humans Vol 100B, A Review of Human Carcinogen: Biological Agents 2012 Lyon, France: International Agency for Research on Cancer

[B6] Dukers NH Rezza G Human herpesvirus 8 epidemiology: what we do and do not know AIDS 2003 17 12 1717 1730 10.1097/00002030-200308150-00001 12891058

[B7] Dedicoat M Newton R Review of the distribution of Kaposi’s sarcoma-associated herpesvirus (KSHV) in Africa in relation to the incidence of Kaposi’s sarcoma Br J Cancer 2003 88 1 1 3 10.1038/sj.bjc.6600745 12556950 PMC2376771

[B8] Biryahwaho B Dollard SC Pfeiffer RM Shebl FM Munuo S Amin MM Hladik W Parsons R Mbulaiteye SM Sex and geographic patterns of human herpesvirus 8 infection in a nationally representative population-based sample in Uganda J Infect Dis 2010 202 9 1347 1353 10.1086/656525 20863232 PMC2949503

[B9] Shebl FM Dollard SC Pfeiffer RM Biryahwaho B Amin MM Munuo SS Hladik W Parsons R Graubard BI Mbulaiteye SM Human herpesvirus 8 seropositivity among sexually active adults in Uganda PLoS One 2012 6 6 e21286 21712983 10.1371/journal.pone.0021286 PMC3119672

[B10] Serraino D Toma L Andreoni M Butto S Tchangmena O Sarmati L Monini P Franceschi S Ensoli B Rezza G A seroprevalence study of human herpesvirus type 8 (HHV8) in eastern and Central Africa and in the Mediterranean area Eur J Epidemiol 2001 17 9 871 876 10.1023/A:1015678312153 12081107

[B11] Engels EA Clark E Aledort LM Goedert JJ Whitby D Kaposi’s sarcoma-associated herpesvirus infection in elderly Jews and non-Jews from New York City Int J Epidemiol 2002 31 5 946 950 10.1093/ije/31.5.946 12435765

[B12] Engels EA Atkinson JO Graubard BI McQuillan GM Gamache C Mbisa G Cohn S Whitby D Goedert JJ Risk factors for human herpesvirus 8 infection among adults in the United States and evidence for sexual transmission J Infect Dis 2007 196 2 199 207 10.1086/518791 17570106

[B13] Mbulaiteye SM Atkinson JO Whitby D Wohl DA Gallant JE Royal S Goedert JJ Rabkin CS Risk factors for human herpesvirus 8 seropositivity in the AIDS Cancer Cohort Study J Clin Virol 2006 35 4 442 449 10.1016/j.jcv.2005.10.010 16414306

[B14] Mbulaiteye SM Biggar RJ Pfeiffer RM Bakaki PM Gamache C Owor AM Katongole-Mbidde E Ndugwa CM Goedert JJ Whitby D Water Engels EA Water, socioeconomic factors, and human herpesvirus 8 infection in Ugandan children and their mothers J Acquir Immune Defic Syndr 2005 38 4 474 479 10.1097/01.qai.0000132495.89162.c0 15764964

[B15] Mbulaiteye SM Pfeiffer RM Engels EA Marshall V Bakaki PM Owor AM Ndugwa CM Katongole-Mbidde E Goedert JJ Biggar RJ Whitby D Detection of kaposi sarcoma-associated herpesvirus DNA in saliva and buffy-coat samples from children with sickle cell disease in Uganda J Infect Dis 2004 190 8 1382 1386 10.1086/424489 15378429

[B16] Mbulaiteye SM Pfeiffer RM Whitby D Brubaker GR Shao J Biggar RJ Human herpesvirus 8 infection within families in rural Tanzania J Infect Dis 2003 187 11 1780 1785 10.1086/374973 12751036

[B17] Butler LM Were WA Balinandi S Downing R Dollard S Neilands TB Gupta S Rotherford GW Mermin J Human Herpesvirus 8 Infection in Children and Adults in a Population-based Study in Rural Uganda J Infect Dis 2011 203 5 625 634 10.1093/infdis/jiq092 21273188 PMC3071279

[B18] Eltom MA Mbulaiteye SM Dada AJ Whitby D Biggar RJ Transmission of human herpesvirus 8 by sexual activity among adults in Lagos, Nigeria AIDS 2002 16 18 2473 2478 10.1097/00002030-200212060-00014 12461423

[B19] Wawer MJ Eng SM Serwadda D Sewankambo NK Kiwanuka N Li C Gray RH Prevalence of Kaposi sarcoma-associated herpesvirus compared with selected sexually transmitted diseases in adolescents and young adults in rural Rakai District, Uganda Sex Transm Dis 2001 28 2 77 81 10.1097/00007435-200102000-00003 11234789

[B20] Malope BI MacPhail P Mbisa G MacPhail C Stein L Ratshikhopha EM Ndhlovu L Sitas F Whitby D No evidence of sexual transmission of Kaposi’s sarcoma herpes virus in a heterosexual South African population AIDS 2008 22 4 519 526 10.1097/QAD.0b013e3282f46582 18301065

[B21] Mbulaiteye SM Biggar RJ Bakaki PM Pfeiffer RM Whitby D Owor AM Katongole-Mbidde E Goedert JJ Ndugwa CM Engels EA Human herpesvirus 8 infection and transfusion history in children with sickle-cell disease in Uganda J Natl Cancer Inst 2003 95 17 1330 1335 10.1093/jnci/djg039 12953087

[B22] Lavreys L Chohan B Ashley R Richardson BA Corey L Mandaliya K Ndinya-Achola JO Kreiss JK Human herpesvirus 8: seroprevalence and correlates in prostitutes in Mombasa, Kenya J Infect Dis 2003 187 3 359 363 10.1086/367703 12552419

[B23] Baeten JM Chohan BH Lavreys L Rakwar JP Ashley R Richardson BA Mandaliya K Bwayo JJ Kreiss JK Correlates of human herpesvirus 8 seropositivity among heterosexual men in Kenya AIDS 2002 16 15 2073 2078 10.1097/00002030-200210180-00013 12370507

[B24] Ministry of Health (MOH) [Uganda] and ORC Macro Uganda HIV/AIDS Sero-Behavioural Survey 2004–2005 2006 Calverton, MD: Ministry of Health and ORC Macro

[B25] Mbulaiteye SM Goedert JJ Transmission of Kaposi sarcoma-associated herpesvirus in sub-Saharan Africa AIDS 2008 22 4 535 537 10.1097/QAD.0b013e3282f4352e 18301068

[B26] de Sanjose S Mbisa G Perez-Alvarez S Benavente Y Sukvirach S Hieu NT Shin HR Anh PT Thomas J Lazcano E Matos E Herrero R Munoz N Molano M Franceschi S Whitby D Geographic variation in the prevalence of Kaposi sarcoma-associated herpesvirus and risk factors for transmission J Infect Dis 2009 199 10 1449 1456 10.1086/598523 19351262

[B27] Dollard SC Butler LM Jones AM Mermin JH Chidzonga M Chipato T Shiboski CH Brander C Mosam A Kiepiela P Hladik W Martin JN Substantial regional differences in human herpesvirus 8 seroprevalence in sub-Saharan Africa: insights on the origin of the “Kaposi’s sarcoma belt” Int J Cancer 2010 127 10 2395 2401 10.1002/ijc.25235 20143397 PMC2895015

[B28] Pfeiffer RM Wheeler WA Mbisa G Whitby D Goedert JJ de The G Mbulaiteye SM Geographic heterogeneity of prevalence of the human herpesvirus 8 in sub-Saharan Africa: clues about etiology Ann Epidemiol 2010 20 12 958 963 10.1016/j.annepidem.2010.07.098 21074111

[B29] Mbulaiteye SM Pfeiffer RM Dolan B Tsang VC Noh J Mikhail NN Abdel-Hamid M Hashem M Whitby D Thomas Strickland G Goedert JJ Seroprevalence and risk factors for human herpesvirus 8 infection, rural Egypt Emerg Infect Dis 2008 14 4 586 591 10.3201/eid1404.070935 18394276 PMC2570936

[B30] Lin CJ Katongole-Mbidde E Byekwaso T Orem J Rabkin CS Mbulaiteye SM Intestinal parasites in Kaposi sarcoma patients in Uganda: indication of shared risk factors or etiologic association AmJTrop Med Hyg 2008 78 3 409 412 18337336

[B31] Wakeham K Webb EL Sebina I Muhangi L Miley W Johnson WT Ndibazza J Elliott AM Whitby D Newton R Parasite infection is associated with Kaposi’s sarcoma associated herpesvirus (KSHV) in Ugandan women Infect Agent Cancer 2011 6 1 15 10.1186/1750-9378-6-15 21962023 PMC3197512

[B32] Johnstone IM Lu AY On Consistency and Sparsity for Principal Components Analysis in High Dimensions J Am Stat Assoc 2009 104 486 682 693 10.1198/jasa.2009.0121 20617121 PMC2898454

[B33] Rabkin CS Schulz TF Whitby D Lennette ET Magpantay LI Chatlynne L Biggar RJ Interassay correlation of human herpesvirus 8 serologic tests. HHV-8 Interlaboratory Collaborative Group J Infect Dis 1998 178 2 304 309 10.1086/515649 9697708

[B34] Mermin J Musinguzi J Opio A Kirungi W Ekwaru JP Hladik W Kaharuza F Downing R Bunnell R Risk factors for recent HIV infection in Uganda JAMA 2008 300 5 540 549 10.1001/jama.300.5.540 18677026

[B35] Bunnell R Opio A Musinguzi J Kirungi W Ekwaru P Mishra V Hladik W Kafuko J Madraa E Mermin J HIV transmission risk behavior among HIV-infected adults in Uganda: results of a nationally representative survey AIDS 2008 22 5 617 624 10.1097/QAD.0b013e3282f56b53 18317003

[B36] Uganda Bureau of Statistics The 2002 Uganda population and housing census, population size and distribution 2006 Kampala: UBS Available from: http://www.ubos.org/onlinefiles/uploads/ubos/pdf%20documents/2002%20CensusPopnSizeGrowthAnalyticalReport.pdf [accessed 15 July 2011]

[B37] Pau CP Lam LL Spira TJ Black JB Stewart JA Pellett PE Respess RA Mapping and serodiagnostic application of a dominant epitope within the human herpesvirus 8 ORF 65-encoded protein J Clin Microbiol 1998 36 6 1574 1577 9620379 10.1128/jcm.36.6.1574-1577.1998 PMC104879

[B38] Spira TJ Lam L Dollard SC Meng YX Pau CP Black JB Burns D Cooper B Hamid M Huong J Kite-Powell K Pellett PE Comparison of serologic assays and PCR for diagnosis of human herpesvirus 8 infection J Clin Microbiol 2000 38 6 2174 2180 10834972 10.1128/jcm.38.6.2174-2180.2000 PMC86757

[B39] Hladik W Dollard SC Mermin J Fowlkes AL Downing R Amin MM Banage F Nzaro E Kataaha P Dondero TJ Pellett PE Lackritz EM Transmission of human herpesvirus 8 by blood transfusion N Engl J Med 2006 355 13 1331 1338 10.1056/NEJMoa055009 17005950

[B40] Rao JNK Scott AJ The analysis of categorical data from complex surveys: Chi-squared test for goodness of fit and independence in two-way tables J Am Stat Assoc 1981 76 221 230 10.1080/01621459.1981.10477633

